# Transcoronary ethanol ablation for incessant ventricular tachycardia: a salvage technique when faced with left ventricular thrombus

**DOI:** 10.1007/s12471-015-0746-x

**Published:** 2015-09-09

**Authors:** A. Frontera, T.W. Johnson, G. Thomas, E. Duncan

**Affiliations:** Bristol Heart Institute, University Hospitals of Bristol NHS Foundation Trust, BS2 8HW Bristol, UK

**Fig. 1:**
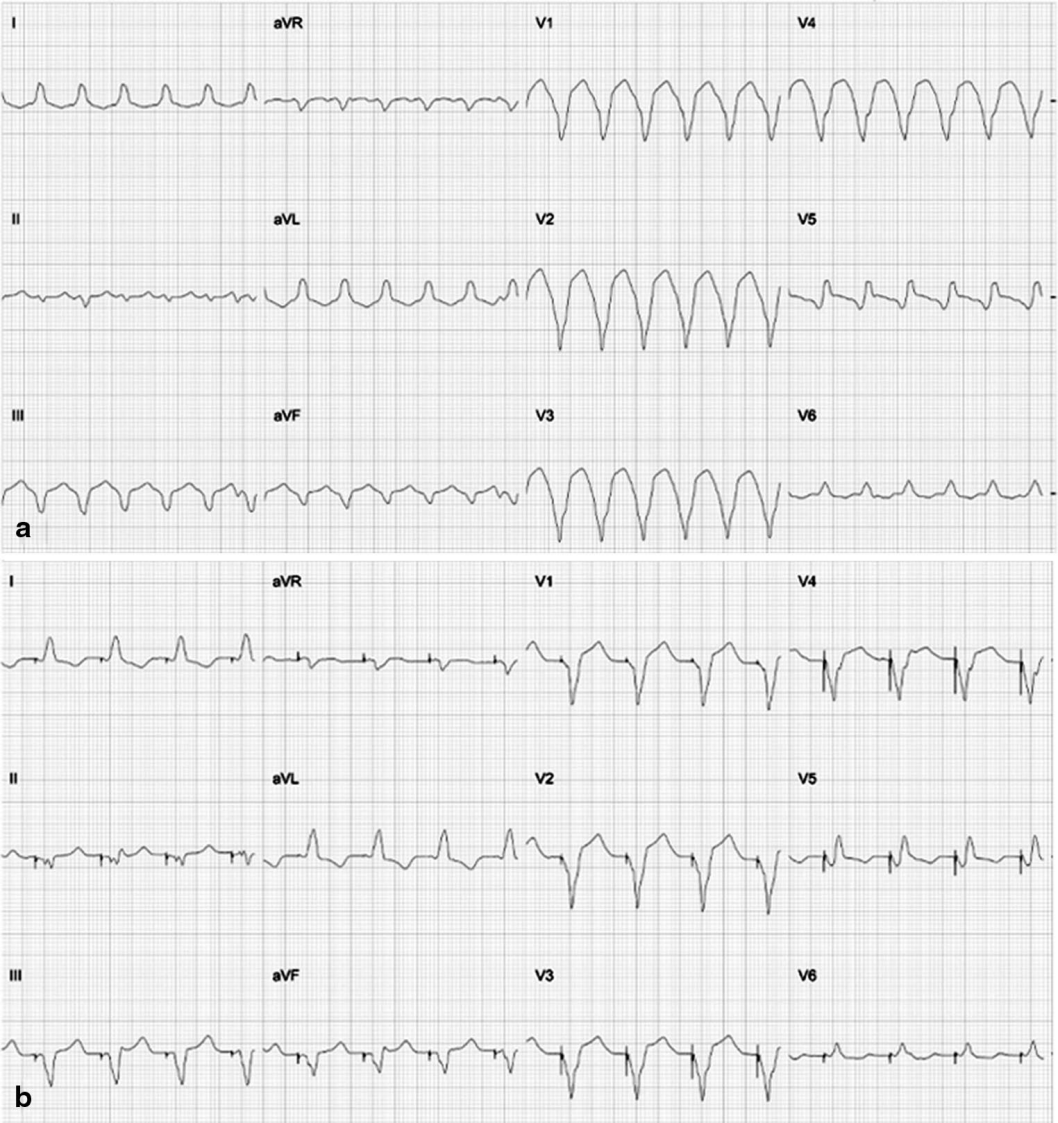
Twelve-lead ECG of ventricular tachycardia (Panel *A*) and at the site of pacemap match (Panel *B*)

**Fig. 2:**
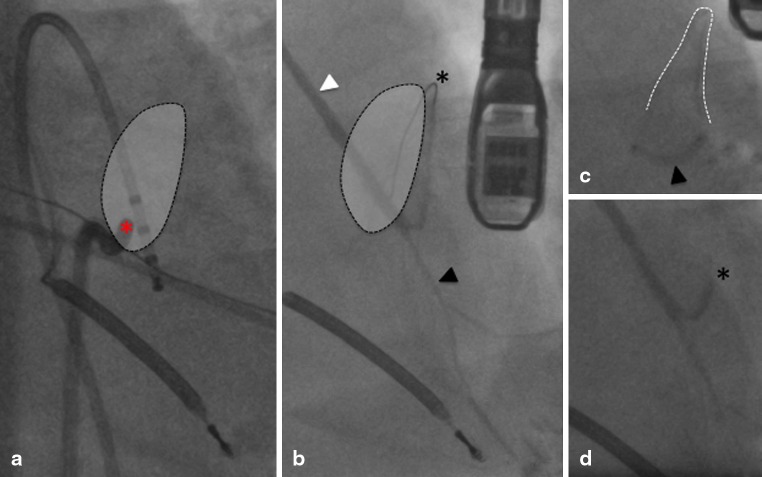
Fluoroscopic images of best pacemap (red asterisk) at VT exit site on RV side of interventricular septum (Panel *A*); patent LAD graft (white arrowhead) and distal LAD (black arrowhead) with interventional wire retrogradely inserted into septal branch of LAD (black asterisk) (Panel *B*); balloon occlusion (black arrowhead) of retrograde limb of LAD (Panel *C*) with final angiographic assessment (Panel *D*). See text for further details

A 77-year-old man with a history of bypass surgery following an anterior myocardial infarction was admitted with a VT storm (Fig. 1A). Coronary angiography revealed occlusion of the left anterior descending (LAD) artery with a patent vein graft to the distal vessel, backfilling the mid-anterior septum. Left ventriculography demonstrated poor left ventricular (LV) systolic function and a calcified apical aneurysm with thrombus (confirmed by contrast echocardiography). LV endocardial mapping was therefore abandoned. Epicardial mapping identified no target. After further shocks and failed medical therapy, endocardial mapping revealed an exit site at the right ventricular septum adjacent to a scar zone and the thrombus within the LV aneurysm (Fig. 2A). The red asterisk marks the site of the best pacemap (Fig. 1B) where the tip of the catheter is on the right side of the interventricular septum. Angiography was performed with the LAD graft (white arrowhead) and the distal LAD (black arrowhead) patent (Fig. 2B). The shaded area denotes the superimposed territory of a mid-LAD septal branch on angiography (Fig. 2A and B). An interventional wire was retrogradely advanced into the septal branch of the LAD (black asterisk) that subtended the territory of VT origin (shaded region). A balloon occluded (black arrowhead) the retrograde limb of the LAD with contrast injection confirming the position (white dotted line) (Panel C, Fig. 2). After injection of a single 2mL aliquot of ethanol (dehydrated alcohol, Martindale) over 3 min, final angiographic assessment confirmed occlusion of the retrograde limb of the LAD (Panel D, Fig. 2). Clinical VT was rendered non-inducible. Follow-up at 15 months confirmed no further VT. Transcoronary ethanol ablation represents a viable salvage technique when faced with LV thrombus.

